# Bryostatin‐1 ameliorated experimental colitis in *Il‐10* ^−/−^ Mice by protecting the intestinal barrier and limiting immune dysfunction

**DOI:** 10.1111/jcmm.14457

**Published:** 2019-06-28

**Authors:** Lugen Zuo, Jing Li, Sitang Ge, Yuanyuan Ge, Mengdi Shen, Yan Wang, Changmin Zhou, Rong Wu, Jianguo Hu

**Affiliations:** ^1^ Department of Gastrointestinal Surgery First Affiliated Hospital of Bengbu Medical College Bengbu China; ^2^ Anhui Key Laboratory of Tissue Transplantation Bengbu Medical College Bengbu China; ^3^ Department of Clinical Laboratory First Affiliated Hospital of Bengbu Medical College Bengbu China; ^4^ Department of Colorectal Surgery The Third Affiliated Hospital of Nanjing University of Chinese Medicine Nanjing China; ^5^ Department of Clinical medicine Bengbu medical college Bengbu China; ^6^ Department of General Surgery Zhongda Hospital, Southeast University Nanjing China

**Keywords:** bryostatin‐1, Crohn's disease, intestinal mucosal immunity, intestinal barrier

## Abstract

Bryostatin‐1 (Bry‐1) has been proven to be effective and safe in clinical trials of a variety of immune‐related diseases. However, little is known about its effect on Crohn's disease (CD). We aimed to investigate the impact of Bry‐1 on CD‐like colitis and determine the mechanism underlying this effect. In the present study, 15‐week‐old male *Il‐10* ^−/− ^mice with spontaneous colitis were divided into positive control and Bry‐1‐treated (Bry‐1, 30 μg/kg every other day, injected intraperitoneally for 4 weeks) groups. Age‐matched, male wild‐type (WT) mice were used as a negative control. The effects of Bry‐1 on colitis, intestinal barrier function and T cell responses as well as the potential regulatory mechanisms were evaluated. We found that the systemic delivery of Bry‐1 significantly ameliorated colitis in *Il‐10* ^−/− ^mice, as demonstrated by decreases in the disease activity index (DAI), inflammatory score and proinflammatory mediator levels. The protective effects of Bry‐1 on CD‐like colitis included the maintenance of intestinal barrier integrity and the helper T cell (Th)/regulatory T cell (Treg) balance. These effects of Bry‐1 may act in part through nuclear factor erythroid 2‐related factor 2 (Nrf2) signalling activation and STAT3/4 signalling inhibition. The protective effect of Bry‐1 on CD‐like colitis suggests Bry‐1 has therapeutic potential in human CD, particularly given the established clinical safety of Bry‐1.

## 1 INTRODUCTION

Crohn's disease (CD) is a chronic, relapsing, progressive disease characterized by immune‐mediated inflammation in the gastrointestinal tract.^1^ The incidence and prevalence of CD is increasing worldwidely.^1^, ^2^ In the absence of aetiological treatment, medical therapy with the purpose of inducing and maintaining remission plays a key role in the management of CD.^3^ Safer and more effective drugs need to be found to compensate for the shortcomings of current medical treatments.^1^, ^3^

Although the pathogenesis of CD remains largely unknown, CD is considered a disease arising from an interaction between genetic and environmental factors.^1^ Over activated T helper cell type 1 (Th1) and T helper cell type 17 (Th17) immune responses contribute to intestinal damage in CD.^4^ Regulatory T cells (Tregs), which are required for controlling excessive inflammation and maintaining immune homeostasis, also play an essential role in the pathogenesis of CD.^5^ Undoubtedly, intestinal mucosal inflammation can indirectly or directly damage the intestinal barrier.^6^ The intestinal epithelium at the interface between the intestinal microbiome/other antigens and the lymphoid tissue associated with the gastrointestinal system plays a critical role in shaping the mucosal immune response.^7^, ^8^ In addition, the inflammatory response often results in continued epithelial injury, which causes erosions and ulcerations. Indirect data suggest that a barrier defect might precede the onset of disease.^7^ This conclusion stems from the fact that even patients with quiescent CD exhibit increased paracellular permeability.^9^ However, there is no answer to the question of whether barrier dysfunction is a cause or consequence of CD. It seems that cross‐talk between the impaired intestinal barrier and abnormal intestinal mucosal immune responses plays an important role in the occurrence and development of CD.

The diseased bowel could be removed by surgery, however, recurrence would still occur after surgery. In fact, CD is a disease with a tendency to relapse for life. However, protecting the intestinal mucosal barrier or regulating intestinal mucosal immunity should be beneficial to CD patients.^10^ Interleukin (IL)‐10 gene‐deficient (*Il‐10* ^−/−^) mice are recognized as a CD animal model with intestinal barrier defects and abnormal intestinal mucosal immunity similar to those in human CD.^11^, ^12^ We have long been committed to the study of how to improve the intestinal barrier and intestinal mucosal immunity in *Il‐10* ^−/− ^mice from multiple perspectives, including inhibiting Th1/Th17 immune responses,^13^ promoting Treg activities,^14^, ^15^ limiting intestinal mucosal epithelial cell apoptosis,^14^, ^16^ protecting intestinal epithelial tight junction (TJ) proteins^17^ and regulating autophagy.^15^ However, we still think more agents, particularly drugs with the potential for rapid clinical application, should be developed to meet CD treatment needs.

Bryostatin‐1 (Bry‐1) is a naturally occurring macrocyclic lactone obtained from the marine bryozoan *Bugula neritina*.^18^ Previous studies have shown that Bry‐1 has a variety of biological activities, including protecting cell TJs, anti‐inflammatory functions and immune regulation.^19^ A recent study indicated that Bry‐1 could promote the differentiation of CD4+ T helper (Th) lymphocytes into Th2 effector cells over Th1 and Th17 effector cells in experimental multiple sclerosis mice.^20^ Furthermore, recent phase IIa clinical trials confirmed that Bry‐1 not only showed good efficacy but also exhibited high safety in the treatment of Alzheimer's disease.^21^, ^22^ These findings suggest Bry‐1 has potential as a therapeutic agent in CD, particularly given its established clinical safety.

In the present study, we investigated the effects of Bry‐1 on spontaneous CD‐like colitis in *Il‐10* ^−/−^ mice and the possible mechanism of these effects with the hope of providing a new therapeutic option for CD.

## 2 MATERIALS AND METHODS

### 2.1 Mice

Our experimental procedures were approved by the Animal Ethics Committee of Bengbu Medical College (Bengbu, China). Wild‐type (WT) mice (C57BL/6J) and *Il‐10* ^−/−^ mice on the C57BL/6J background were obtained from the Jackson Laboratory and were maintained in a specific pathogen‐free (SPF) environment at the Animal Center of Bengbu Medical College (Bengbu, China). All mice were housed in plastic‐bottomed, wire‐lidded cages and kept at 25°C with a 12‐hour light/dark cycle. The mice had free access to water and were fed regular mouse chow. As reported previously, the *Il‐10* ^−/− ^mice consistently developed colitis at 15 weeks of age when housed in the SPF environment. 23

### 2.2 Drug administration protocol

Fifteen‐week‐old male *Il‐10* ^−/− ^mice with spontaneous enteritis were divided into positive control (IL‐10^−/−^) and *Bry‐1*‐treated groups (Bry‐1). Age‐matched male WT mice were used as negative controls (WT). Each group in this study contained eight mice. Bry‐1 with a purity ≥99% (solid) was purchased from Sigma‐Aldrich (Cat#: B7431, St. Louis, MO). A working Bry‐1 solution was prepared by initially dissolving Bry‐1 in 100% ethanol and then further diluting the mixture to 20% ethanol and 1% DMSO in phosphate‐buffered saline (PBS). The final concentration of Bry‐1 was 10 μg/mL. The treatment group received an intraperitoneal (ip) injection of 30 μg/kg Bry‐1 every other day for 4 weeks, and the control groups received an equal volume of vehicle control (20% ethanol and 1% DMSO in PBS) every other day for 4 weeks, as previously described.^20^ After 4 weeks, the mice were killed under anaesthesia. The entire colon was collected, carefully rinsed with PBS and prepared for subsequent examination.

### 2.3 Colitis symptom assessment

Each *Il‐10* ^−/− ^mouse was scored weekly with the inflammatory bowel disease activity index (DAI) using the numerical system described by Spencer et al^23^ Briefly, the DAI was calculated by scoring 1 point for the appearance of each of the following characteristics: ruffled fur, occult faecal blood, rectal prolapse <1 mm and soft stool, with an additional point for diarrhoea or severe rectal prolapse >1 mm. The DAI was calculated on a 6‐point (0‐5) scale.

### 2.4 Histological analysis

Tissue from the colon was routinely stained with haematoxylin and eosin (H&E) and analysed for morphological changes. The intestinal inflammation grading was performed as previously described.^24^ Briefly, intestinal inflammation was scored on a scale of 0‐4 as follows: 0, no inflammation; 1, infiltration of a modest number of cells into the lamina propria; 2, infiltration of mononuclear cells leading to the separation of the crypts and mild mucosal hyperplasia; 3, massive infiltration of inflammatory cells accompanied by disrupted mucosal architecture, loss of goblet cells and marked mucosal hyperplasia; and 4, all of the above features plus crypt abscesses or ulceration. All sections were scored by two independent histologists who were blinded to the treatment group.

### 2.5 Intestinal permeability in vivo

As we previously described,^25^ the mice were fasted for 4 hours and then administered fluorescein isothiocyanate (FITC)‐dextran (4 kDa; Sigma‐Aldrich, Cat#: FD4, St. Louis, MO) by gavage at a dose of 600 mg/kg. After 4 hours, the mice were killed and bled by cardiac puncture. The serum was isolated using centrifugation, and the serum FITC levels were evaluated using fluorometry.

### 2.6 Bacterial translocation

As we previously described,^25^ using aseptic techniques, tissue samples from the mesenteric lymph nodes (MLN) and liver were taken for bacteriological cultures. Two samples for each histologic type were taken for culture respectively. The collected tissue samples were weighed, and 0.1 g of each sample was homogenized in a tissue grinder with 0.9 mL of sterile saline. The homogenates were diluted, and 100 μL dilutions were taken and cultured on MacConkey's agar (Sigma‐Aldrich, Cat#: M7408, St. Louis, MO) at 37°C for 24 hours. Bacterial growth on the plates was expressed as colony‐forming units/g of tissue. The culture result was considered to be positive when more than 10^2^ colony‐forming units/g of tissue were found.^26^

### 2.7 Transmission electron microscopy (TEM) of TJs

Consistent with our procedures in previous report,^25^ sections of colon (2 mm) were fixed for 2 hours in 4% buffered glutaraldehyde. The sections were cut into smaller pieces, after fixed in 1% osmium tetroxide (OsO_4_), sequentially dehydrated through graded alcohols, infiltrated with Epon 812 and then embedded in resin. Thin sections were cut, stained with uranyl acetate and lead citrate and examined with a Hitachi H‐600 transmission electron microscope (Hitachi) operated at 75 kV at a magnification of 20 000×. TJs were considered to have increased permeability when the electron‐dense marker penetrated the junctional complex.

### 2.8 Western blot analysis

Whole‐cell and nuclear protein extracts were prepared from tissue homogenates as previously described,^27^ and Western blot analysis was conducted as previously described.^28^ Briefly, the protein extracts from the tissue homogenate were separated by SDS‐PAGE and transferred to PVDF membranes for immunoblotting. The primary antibodies used were 1:1 000 dilutions of rabbit polyclonal antibodies against Claudin‐1, Occludin, Bcl‐2, Bax, cleaved Caspase‐3, nuclear factor erythroid 2‐related factor 2 (Nrf2; Abcam, Cat#: ab62352, Cambridge, MA, UK), heme oxygenase‐1 (HO‐1; Abcam, Cat#: ab13243), phosphorylated signal transducer and activator of transcription 3 (p‐STAT3; Abcam, Cat#: ab32143), STAT3 (Abcam, Cat#: ab119352), p‐STAT4 (Abcam, Cat#: ab28815), STAT4 (Abcam, Cat#: ab235946,) and β‐actin (Abcam, Cat#: ab8226). The Western blot analyses were performed with a horseradish peroxidase (HRP)‐conjugated secondary antibody at a 1:2000 dilution (Cell Signaling Technology, Cat#: 7075, Beverly, MA) using enhanced chemiluminescence Western blotting detection reagents (Millipore, Cat#: WBULS0500, MA). Protein quantification was performed via optical density methods using ImageJ software (National Institutes of Health, USA). The results are presented as the relative density of each experimental band with respect to the density of the β‐actin band normalized to the mean value of the *Il‐10* ^−/−^ or WT group.^29^

### 2.9 Terminal deoxynucleotidyl transferase‐mediated dUTP nick end labelling (TUNEL) staining

Immunofluorescent TUNEL staining was performed to measure apoptosis in paraffin‐embedded sections using an In Situ Cell Death Detection kit according to the manufacturer's instructions (Roche, Cat#:11684795910，Indianapolis, IN) as previously reported.^30^ Nuclei were stained with 4,6’‐diamidino‐2‐phenylindole (DAPI) to count the total number of cells per crypt. A minimum of 10 crypts with normal morphology were counted per section.

### 2.10 Flow cytometry

T cell responses were analysed by flow cytometry as previously described.^29^ Antibodies specific for Foxp3 (intracellular staining, eBioscience, Cat#: 12‐4777‐42, San Diego, CA), CD4 (eBioscience, Cat#: 11‐0041‐81), and CD25 (eBioscience, Cat#:17‐0259‐42) were used to analyse the proportion of Tregs in the splenocyte and MLN cell populations. The analyses were performed on a FACSCalibur flow cytometer (BD Biosciences, San Diego, CA), and the data were analysed using FlowJo V10 software. Splenocytes and MLN cells were incubated at 2 × 10^6^ cells/mL in 48‐well plates and stimulated with a cell stimulation cocktail (2 μL/well; eBioscience) for 2 hours. Then, 1 μL of Brefeldin A (eBioscience, Cat#: 00‐4506‐51) was added for another 4 hours. After the cells were washed with FACS buffer (PBS, 2% heat‐inactivated foetal bovine serum (FBS), and 0.09% sodium azide), they were stained for surface markers with anti‐CD4 (eBioscience, Cat#: 11‐0041‐81) and anti‐CD3e antibodies (eBioscience, Cat#:17‐0036‐42) for 30 minutes at 4°C, fixed and permeabilized with IC fixation buffer and permeabilization buffer (eBioscience, Cat#: 00‐8222‐49) for 30 minutes at 4°C and incubated with anti‐interferon (IFN)‐γ (eBioscience, Cat#:12‐7311‐81) or anti‐IL‐17A antibodies (eBioscience, Cat#: 12‐9171‐82) for 1 hour at 4°C. The raw sample data were collected using a FACSCalibur flow cytometer (BD Biosciences), and the data were analysed using FlowJo V10 software.

### 2.11 Enzyme‐linked immunosorbent assay (ELISA)

IL‐17A (R&D Systems, Cat#: PM1700, Emeryville, CA), IFN‐γ (R&D Systems Cat#: PMIF00) and TNF‐α (R&D Systems, Cat#: PMTA00B) expression levels in the intestine were determined by ELISAs. Briefly, intestinal tissue was homogenized in 1 mL of normal saline. Then, the homogenates were centrifuged at 3000 rpm at 4°C for 30 minutes, and the supernatant was stored at −80°C until analysis.

### 2.12 Quantitative real‐time PCR (qRT‐PCR) analysis

The *Ifn‐γ*, *Tnf‐α*, *Il‐17A*, *Nrf2* and *HO‐1* mRNA levels were measured using qRT‐PCR analysis as previously described.^31^ Briefly, total RNA was extracted from the tissue homogenate using TRIzol reagent (Life Technologies, Carlsbad, CA), and the oligo (dT)‐primed complementary DNA was made by reverse transcription of the purified RNA. The transcript abundance of the genes of interest was measured using a qRT‐PCR assay with Synergy Brands (SYBR) Green detection (Applied Biosystems, Carlsbad, CA). All reactions were independently repeated at least twice to ensure the reproducibility of the results. The primer sequences used are listed in Table 1. The expression levels of each gene were normalized to the level of GAPDH gene expression to yield relative expression values.

**Table 1:** Primer sequences (5′ to 3′)

Gene name	Forward primer	Reverse primer
*Ifn‐γ*	ACAGCAAGGCGAAAAAGGATG	TGGTGGACCACTCGGATGA
*Tnf‐α*	CAGGCGGTGCCTATGTCTC	CGATCACCCCGAAGTTCAGTAG
*Il‐17A*	TCTCAGGCTCCCTCTTCAG	GACTCTCCACCGCAATGA
*Nrf2*	CGAGATATACGCAGGAGAGGTAAGA	GCTCGACAATGTTCTCCAGCTT
*HO‐1*	CTCCCTGTGTTTCCTTTCTCTC	GCTGCTGGTTTCAAAGTTCAG
*Gapdh*	AGGTCGGTGTGAACGGATTTG	TGTAGACCATGTAGTTGAGGTCA

### 2.13 Immunofluorescence

Immunofluorescence was assessed as described previously.^27^ Colonic segments were immediately removed, washed with PBS, mounted in embedding medium, and stored at −80°C until use. Frozen sections were cut at 10 μm and mounted on slides. Nonspecific background signals were blocked by incubation with 5% bovine serum albumin plus 5% newborn bovine serum in PBS for 30 minutes at room temperature. The sections were incubated with rabbit polyclonal antibodies against occludin (Abcam, Cat#: ab216327, Cambridge, MA, UK) and claudin‐1 (Abcam, Cat#: ab15098, Cambridge, MA, UK) at 4°C overnight. The sections were probed with the appropriate FITC‐conjugated secondary IgG antibodies. The nuclei were counterstained with DAPI. Slides incubated without any primary antibody were used as negative controls. Confocal analysis was performed with a confocal scanning microscope (Leica Microsystems, Heidelberg GmbH, Mannheim, Germany).

### 2.14 Immunohistochemical analysis

The intestinal levels of p‐STAT3 and p‐STAT4 were determined by immunohistochemical analysis as previously described.^32^ Briefly, 5 µm paraffin sections were deparaffinized, rehydrated, submerged in antigen retrieval solution, blocked with normal goat serum for 30 minutes, and incubated at 4°C overnight with a rabbit polyclonal antibody against p‐STAT3 (1:1000; Abcam, Cat#: ab32143) or p‐STAT4 (1:1000; Abcam, Cat#: ab28815). The sections were then incubated for 30 minutes at room temperature with biotinylated goat anti‐rabbit IgG (H + L) immune serum as the secondary antibody and were subsequently incubated with avidin‐biotin complexes (ABC) coupled to peroxidase (Beyotime, Cat#: A0286, Haimen, China). The antigen‐antibody complexes were visualized using 3,3’‐diaminobenzidine (DAB kit, Cat#: P0203, Beyotime), and the sections were dehydrated and mounted. The negative control sections were prepared with the same procedure except the primary antibody was not added. Ten fields from each tissue section were randomly selected to quantify p‐STAT3 and p‐STAT4 expression.

### 2.15 Statistical analysis

The data analyses were performed using Statistical Package for Social Sciences (SPSS; SPSS Inc, Chicago, IL) version 23.0. Continuous, normally distributed data are presented as the mean ± standard deviation (SD). Unpaired *t *tests were used to compare data between two groups. The binary and categorical data were compared using chi‐squared tests to produce the contingency tables. Fisher's exact test was performed when the sample number was ≤5. All tests were two‐sided. A *P* value <0.05 was considered statistically significant.

## 3 RESULTS

### 3.1 Bry‐1 ameliorated experimental colitis in *Il‐10^−/−^* mice

**Figure 1:**
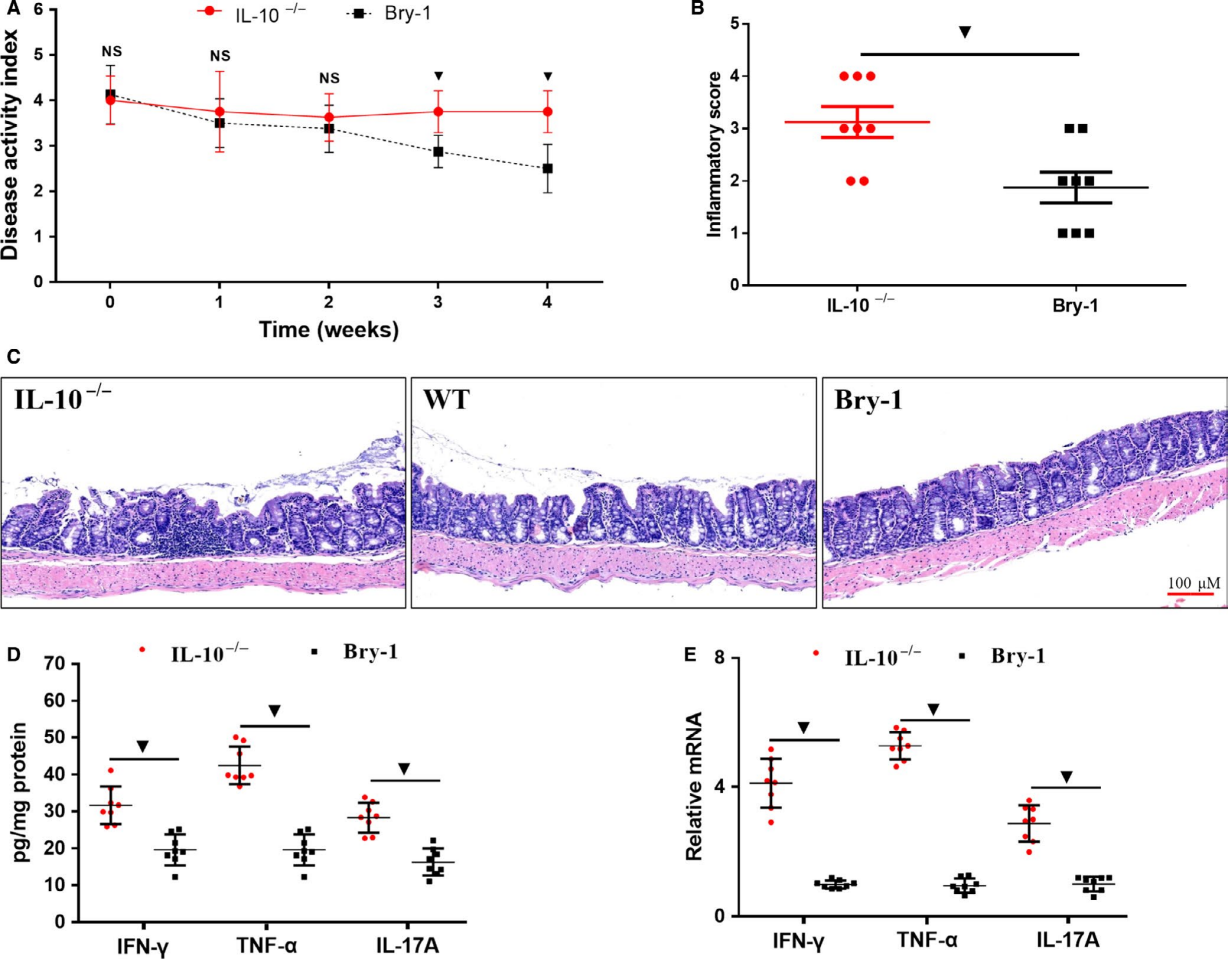
Systemic delivery of Bry‐1 ameliorated experimental colitis in *Il‐10* ^−/− ^mice. (A) Bry‐1‐treated *Il‐10* ^−/− ^mice showed lower mean disease activity index values than untreated *Il‐10* ^−/− ^mice beginning at the third week after drug administration. (C) The systemic delivery of Bry‐1 significantly improved the histological manifestations of chronic colitis (representative H&E staining; scale bar: 100 μmol/L). The histological inflammation score (B) was clearly improved in tissue from Bry‐1‐treated *Il‐10* ^−/−^mice compared with that from untreated *Il‐10* ^−/− ^mice. Both the protein (D) and mRNA (E) levels of IFN‐γ, TNF‐α and IL‐17A were significantly lower in the colonic tissue from Bry‐1‐treated *Il‐10* ^−/− ^mice than that from untreated *Il‐10* ^−/− ^mice. Bry‐1, Bryostatin‐1; WT, wild‐type; IL‐17A, interleukin 17A; IFN‐γ, interferon‐γ; TNF‐α, tumour necrosis factor‐α; and NS, no significance. At least three independent experiments with six to eight mice in each group were performed, with one representative experiment is shown. The data are expressed as the mean ± SD. ^▼^ *P* < 0.05

The Bry‐1‐treated *Il‐10* ^−/−^ mice showed lower mean DAI values than the untreated *Il‐10* ^−/−^ mice beginning at the third week after drug administration (Figure 1A). In addition, the tissue histological inflammation score for the Bry‐1‐treated mice was significantly decreased compared with that for the untreated *Il‐10* ^−/−^ mice (Figure 1B). Moreover, the levels of inflammatory factors (IFN‐γ, TNF‐α and IL‐17A) in the intestinal tissues were significantly decreased in the Bry‐1‐treated *Il‐10* ^−/−^ mice compared with the untreated *Il‐10* ^−/−^ mice at both the protein and mRNA levels (Figure 1C‐D).

### 3.2 Bry‐1 treated *Il‐10^−/−^* mice showed improved disruption of intestinal mucosal epithelial tight junction proteins than the untreated *Il‐10^−/−^* mice

**Figure 2:**
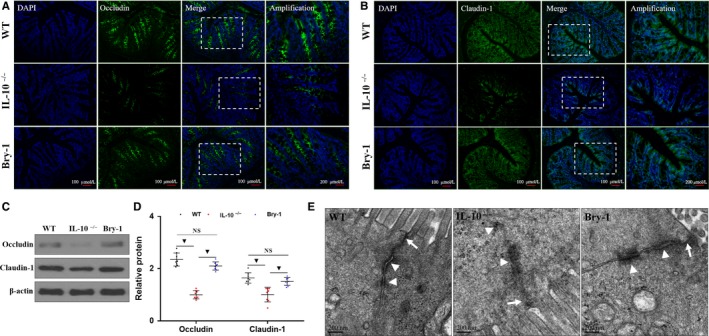
Bryostatin‐1 treated *Il‐10* ^−/−^ mice showed improved disruption of intestinal mucosal epithelial tight junction proteins than the untreated *Il‐10* ^−/−^ mice. Intestinal mucosal epithelial tight junction (TJ) protein (green, occludin and claudin‐1) and DAPI staining (blue), merged TJ protein and DAPI staining as well as amplified merged TJ protein and DAPI staining images are presented (A and B). Western blot analysis showed that the expression of claudin‐1 and occludin in the intestinal mucosa was increased significantly in Bry‐1‐treated *Il‐10* ^−/− ^mice compared with untreated *Il‐10* ^−/− ^mice, and the expression in the Bry‐1‐treated mice was similar to that in WT mice (C and D). The ultrastructural morphology of the TJ structures of intestinal villi was damaged in *Il‐10* ^−/−^ mice, and the damaged was characterized by decreased amounts of electron‐dense materials in the TJs (E, white arrows) and abnormal desmosomes (E, white arrowheads). These damaged TJ structures were improved in Bry‐1‐treated *Il‐10* ^−/−^ mice. Bry‐1, Bryostatin‐1; WT, wild‐type; TJ, tight junction; and NS, no significance. At least three independent experiments with six to eight mice in each group were performed, with one representative experiment is shown. The data are expressed as the mean ± SD. ^▼^ *P* < 0.05

The impaired intestinal mucosal barrier is one of the major pathological changes of CD, and TJ proteins contribute to barrier structure. We assessed the changes in key TJ proteins in response to treatment by immunofluorescence and found that the expression of claudin‐1 and occludin was significantly increased in the Bry‐1‐treated *Il‐10* ^−/−^ mice compared with the untreated *Il‐10* ^−/−^ mice, and the expression levels in the Bry‐1‐treated *Il‐10* ^−/−^ mice were similar to those in the WT mice (Figure 2A,B). This result was confirmed by Western blot analysis (Figure 2C,D). TEM was performed to provide a more direct analysis of the TJ structures of intestinal mucosal epithelial cells at the submicroscopic level (Figure 2E). We found that the ultrastructural morphology of the TJs in intestinal villi was damaged in the untreated *Il‐10* ^−/−^ mice, and this damage was characterized by decreased amounts of electron‐dense materials in the TJs (Figure 2E, white arrows) and abnormal desmosomes (Figure 2E, white arrowheads). However, the damaged TJ structure was improved in the Bry‐1‐treated *Il‐10* ^−/−^ mice. These results indicated that treatment with Bry‐1 could ameliorate the damage to the intestinal barrier structure.

### 3.3 Bry‐1 treated *Il‐10^−/−^* mice show decreased intestinal permeability than the untreated *Il‐10^−/−^* mice

**Figure 3:**
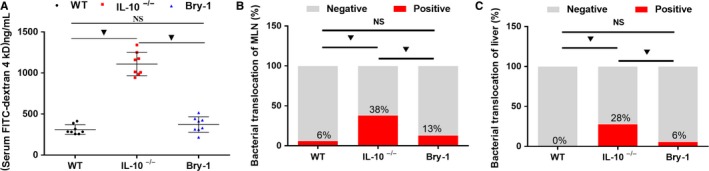
Bryostatin‐1 treated *Il‐10* ^−/−^ mice show decreased intestinal permeability than the untreated *Il‐10* ^−/−^ mice. The evaluation of intestinal permeability in vivo showed that Bry‐1‐treated *Il‐10* ^−/−^ mice had lower levels of serum dextran conjugates than *Il‐10* ^−/−^ mice, and the levels in the Bry‐1‐treated mice were similar to those in WT mice (A). The rates of bacterial translocation to the MLN (B) and liver (C) were lower in Bry‐1‐treated mice than *Il‐10* ^−/−^ mice, and the levels in the Bry‐1‐treated mice were similar to those in WT mice. Bry‐1, Bryostatin‐1; WT, wild‐type; MLN, mesenteric lymph nodes; and NS, no significance. At least three independent experiments with six to eight mice in each group were performed, with one representative experiment is shown. The data are expressed as the mean ± SD. ^▼^ *P* < 0.05

**Figure 4:**
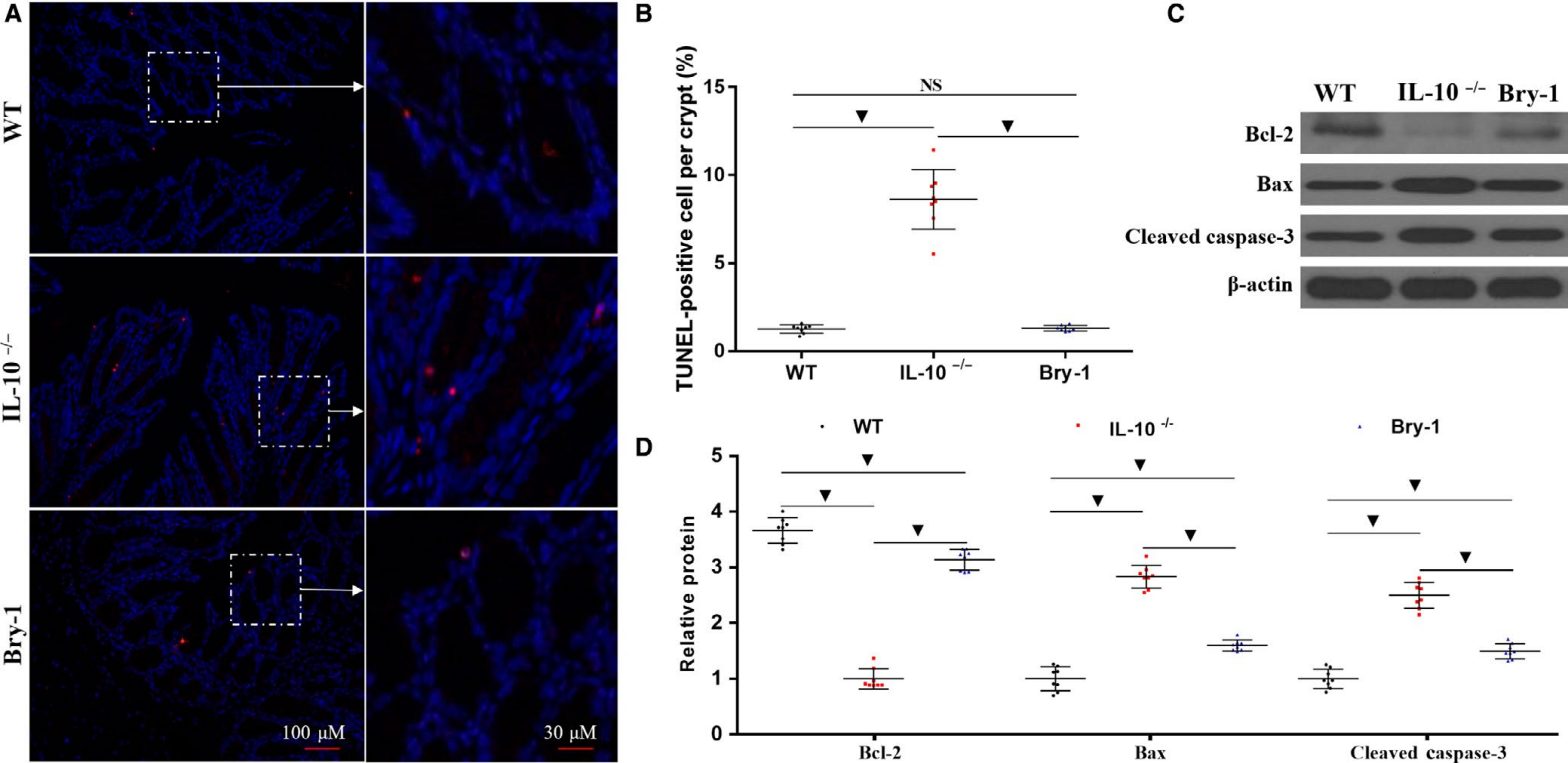
Bryostatin‐1 treated *Il‐10* ^−/−^ mice show less apoptotic intestinal epithelial cells than the untreated *Il‐10* ^−/−^ mice. (A and B) Bry‐1‐treated *Il‐10* ^−/−^ mice exhibited fewer TUNEL‐positive cells per crypt than untreated *Il‐10* ^−/−^ mice, and the number of TUNEL‐positive cells in the Bry‐1‐treated mice was similar to that in WT mice. (C and D) The protein level of the antiapoptotic factor Bcl‐2 was increased in Bry‐1‐treated *Il‐10* ^−/−^ mice compared with untreated *Il‐10* ^−/−^ mice; however, the level in the Bry‐1‐treated mice was still lower than that in WT mice. In contrast, compared to the untreated *Il‐10* ^−/−^ mice, the Bry‐1‐treated *Il‐10* ^−/−^ mice showed a significant decrease in Bax and cleaved caspase‐3 expression, but the expression in the Bry‐1‐treated mice was still higher than that in the WT mice. Bry‐1, Bryostatin‐1; WT, wild‐type; and NS, no significance. At least three independent experiments with six to eight mice in each group were performed, with one representative experiment is shown. The data are expressed as the mean ± SD. ^▼^ *P* < 0.05

We evaluated intestinal permeability in vivo. As shown in Figure 3A, the Bry‐1‐treated mice had lower levels of serum dextran conjugates than the untreated *Il‐10* ^−/−^ mice and levels similar to those of the WT mice. Increased intestinal permeability leading to bacterial translocation plays an important role in the aetiology of human CD.^30^ Bacterial cultures were generated from the MLN and liver as shown in Figure 4. The rates of bacterial translocation to the MLN (Figure 3B) and liver (Figure 3C) in the Bry‐1‐treated mice were lower than those in the untreated *Il‐10* ^−/−^ mice and similar to those in the WT mice. These results combined with the abovementioned findings demonstrated that the intestinal barrier structure and function could be maintained with Bry‐1 treatment in a CD animal model.

### 3.4 Bry‐1 treated *Il‐10^−/−^* mice show less apoptotic intestinal epithelial cells than the untreated *Il‐10^−/−^* mice

Intestinal epithelial cell apoptosis is one of the important factors contributing to the damage to the intestinal barrier in human CD.^33^ Therefore, we examined intestinal epithelial cell death by TUNEL staining. As shown in Figure 4A,B, the Bry‐1‐treated *Il‐10* ^−/−^ mice exhibited fewer TUNEL‐positive cells per crypt than the untreated *Il‐10* ^−/−^ mice, and the number of TUNEL‐positive cells per crypt in the Bry‐1‐treated mice was similar to that in the WT mice. The protein levels of the antiapoptotic factor Bcl‐2 were increased in the Bry‐1‐treated *Il‐10* ^−/−^ mice compared with the untreated *Il‐10* ^−/−^ mice; however, the levels in the Bry‐1‐treated mice were still lower than those in the WT mice (Figure 4C,D). In contrast, compared to the untreated *Il‐10* ^−/−^ mice, the Bry‐1‐treated *Il‐10* ^−/−^ mice showed significant decreases in the Bax and cleaved caspase‐3 levels, but the levels in the Bry‐1‐treated mice were still higher than those in the WT mice (Figure 4C,D). These results indicated that the protective effect of Bry‐1 on *Il‐10* ^−/−^ mice was at least partly mediated through preventing intestinal epithelial cell apoptosis.

### 3.5 Antiapoptotic effect of Bry‐1 on *Il‐10^−/−^* mice may be partly mediated by activating Nrf2 signalling

**Figure 5:**
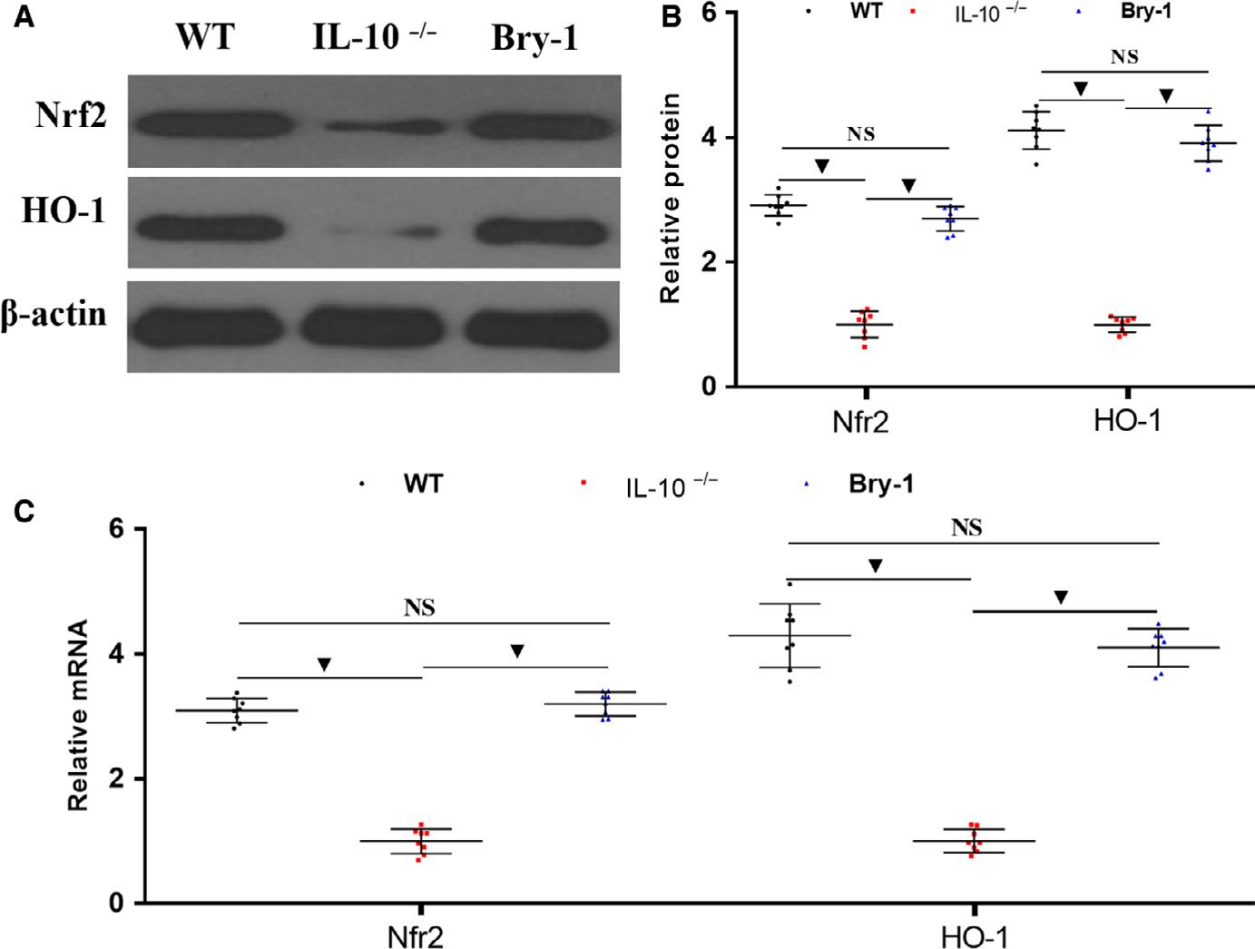
Antiapoptotic effect of Bryostatin‐1 on *Il‐10* ^−/−^ mice may be partly mediated by activating Nrf2 signalling. (A and B) The levels of both Nrf2 and its downstream factor HO‐1 were significantly decreased in Bry‐1‐treated *Il‐10* ^−/−^ mice compared to untreated *Il‐10* ^−/−^ mice, and the levels in the Bry‐1‐treated mice were similar to those in WT mice. These results were confirmed by PCR (C). Bry‐1, Bryostatin‐1; WT, wild‐type; and NS, no significance. At least three independent experiments with six to eight mice in each group were performed, with one representative experiment is shown. The data are expressed as the mean ± SD. ^▼^ *P* < 0.05

We are interested in the antiapoptotic mechanism of Bry‐1, and recently published research showing that the Nrf2 signal is an important antiapoptotic signal.^34^, ^35^, ^36^ As shown in Figure 5, the levels of both Nrf2 and its downstream factor HO‐1 were significantly increased in the Bry‐1‐treated *Il‐10* ^−/−^ mice compared to the untreated *Il‐10* ^−/−^ mice, and the levels in Bry‐1‐treated *Il‐10* ^−/−^ mice were similar to those in the WT mice (Figure 5A,B). These results were confirmed by PCR (Figure 5C). These data indicated that Nrf2 signalling activation may be one of the antiapoptotic mechanisms of Bry‐1.

### 3.6 Bry‐1 treated *Il‐10^−/−^* mice show lower Th1 and Th17 responses than the untreated *Il‐10^−/−^* mice

**Figure 6:**
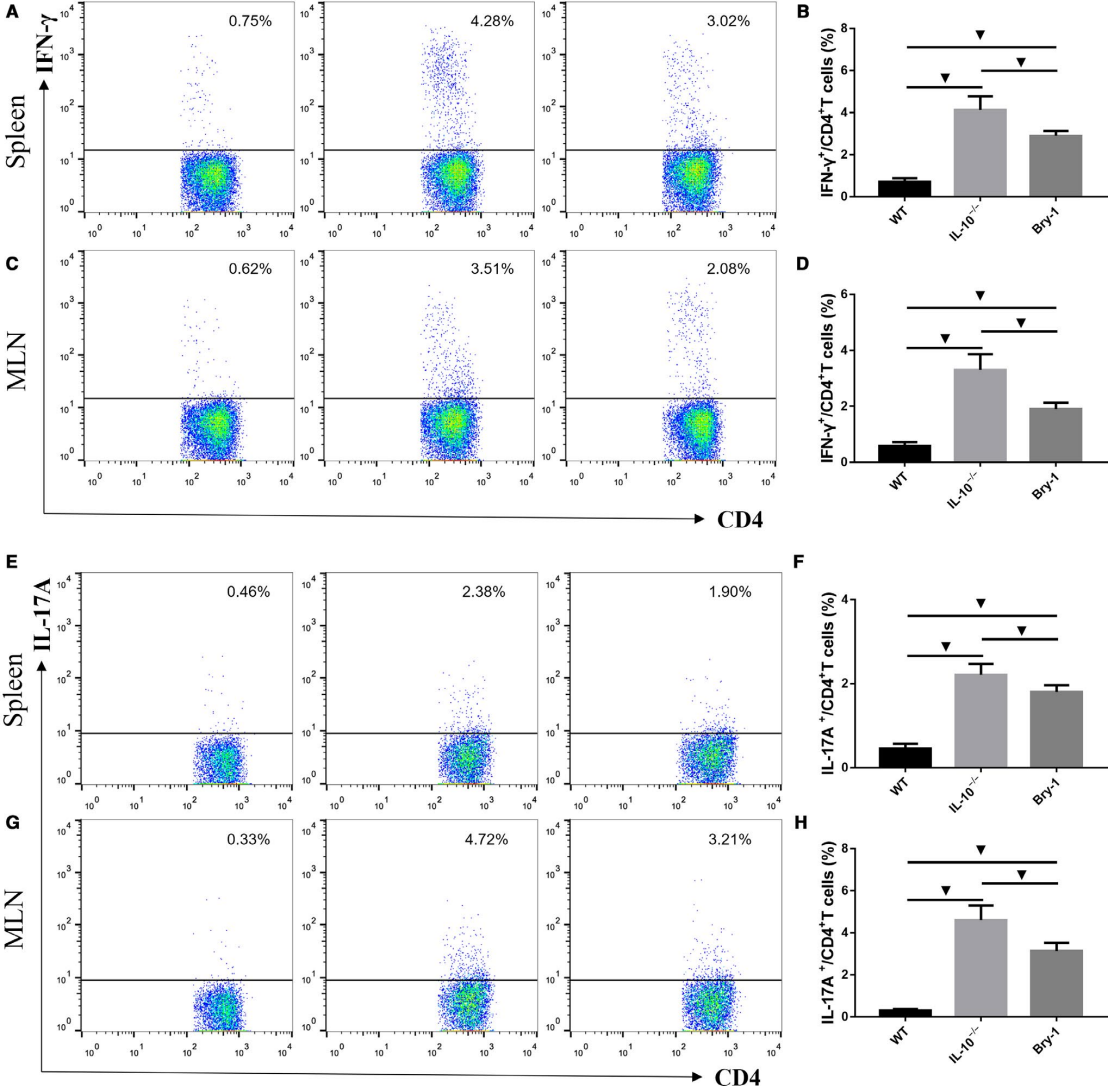
Bryostatin‐1 treated *Il‐10* ^−/−^ mice show lower Th1 and Th17 responses than the untreated *Il‐10* ^−/−^ mice. Intracellular cytokine staining and flow cytometric analysis demonstrated a significant decrease in the proportion of IFN‐γ^+ ^CD4^+^ T cells in Bry‐1‐treated mice compared with untreated *Il‐10* ^−/−^ mice in the spleen (A and B) and MLN (C and D); however, these proportions were still higher than those in WT mice. (E and F) A significant decrease in the proportion of IL‐17A^+ ^CD4^+^ T cells was observed in Bry‐1‐treated mice compared with untreated *Il‐10* ^−/−^ mice in both the spleen (E and F) and MLN (G and H), but these T lymphocyte proportions were still higher than those in WT mice. Bry‐1, Bryostatin‐1; WT, wild‐type; MLN, mesenteric lymph nodes; and NS, no significance. At least three independent experiments with six to eight mice in each group were performed, with one representative experiment is shown. The data are expressed as the mean ± SD. ^▼^ *P* < 0.05

The inhibition of Th1 and Th17 immune responses has long been the approach to treat human CD. Therefore, we performed intracellular cytokine staining to confirm the effect of Bry‐1 on Th1 and Th17 cells in *Il‐10* ^−/−^ mice. As shown in Figure 6, intracellular cytokine staining and flow cytometric analysis demonstrated a significant decrease in the proportion of IFN‐γ^+ ^CD4^+^ T cells in the Bry‐1‐treated mice compared with the untreated *Il‐10* ^−/−^ mice in the spleen (Figure 6A,B) and MLN (Figure 6C,D); however, these proportions were still higher than those in the WT mice. In addition, the flow cytometric data showed a significant decrease in the proportion of IL‐17A^+ ^CD4^+^ T cells in the Bry‐1‐treated mice compared with the untreated *Il‐10* ^−/−^ mice in both the spleen (Figure 6E,F) and the MLN (Figure 6G,H), but these T lymphocyte proportions were still higher than those in the WT mice. These results confirmed that the protective role of Bry‐1 in experimental colitis may be partly mediated by suppressing Th1 and Th17 responses.

### 3.7 Bry‐1 treated *Il‐10^−/−^* mice show higher Treg responses than the untreated *Il‐10^−/−^* mice

**Figure 7:**
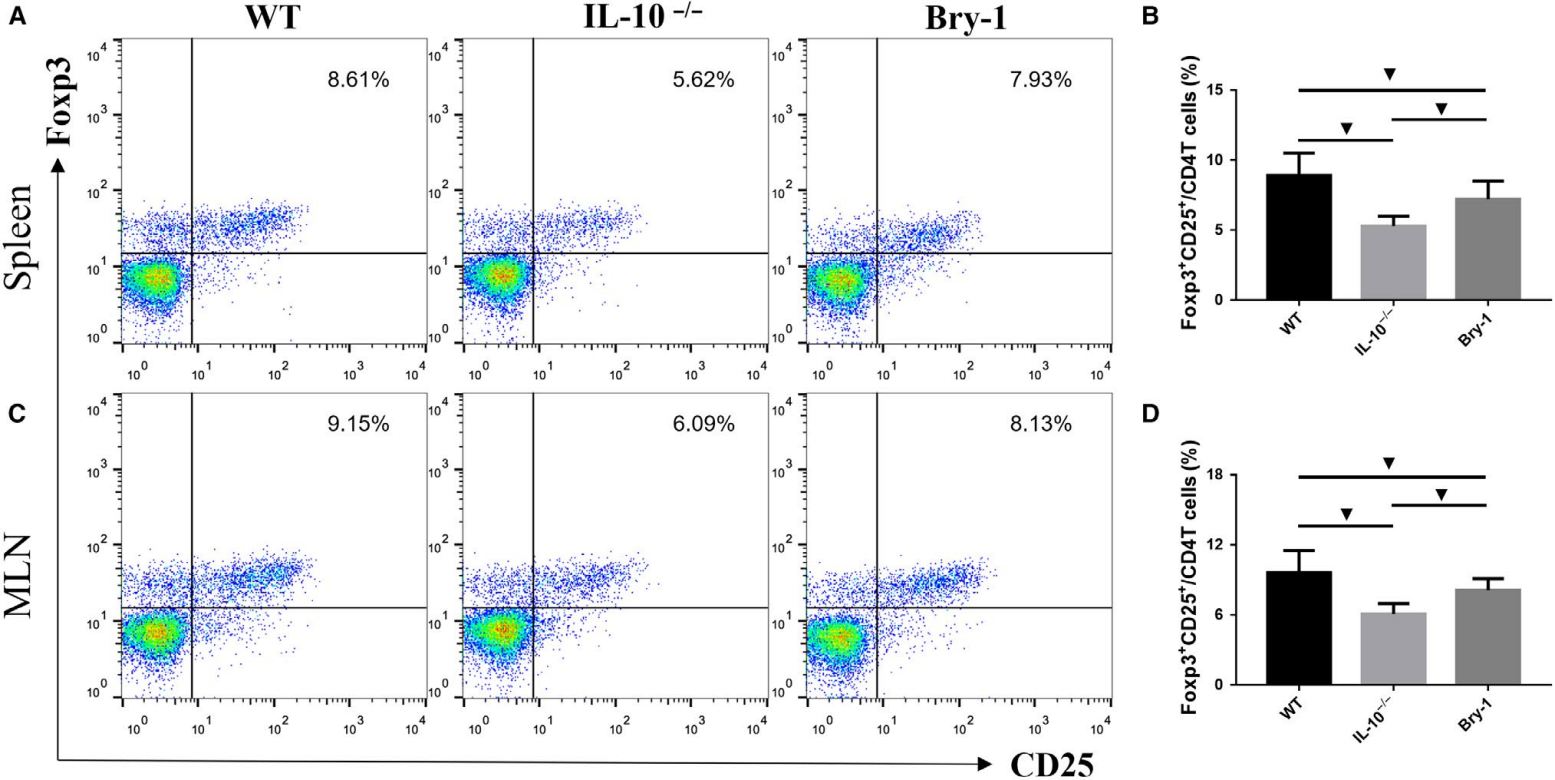
Bryostatin‐1 treated *Il‐10* ^−/−^ mice show higher Treg responses than the untreated *Il‐10* ^−/−^ mice. Bry‐1‐treated *Il‐10* ^−/− ^mice showed lower response of CD4^+ ^CD25^+ ^Foxp3^+ ^T cells (Tregs) when compared with WT mice, the Treg proportion in the Bry‐1‐treated *Il‐10* ^−/− ^mice was significantly higher than that in untreated *Il‐10* ^−/− ^mice in both the spleen (A and B) and the MLN (C and D). Bry‐1, Bryostatin‐1; WT, wild‐type; MLN, mesenteric lymph nodes; and NS, no significance. At least three independent experiments with six to eight mice in each group were performed, with one representative experiment is shown. The data are expressed as the mean ± SD. ^▼^ *P* < 0.05

Tregs have a suppressive effect on Th1 and Th17 responses. We further examined CD4^+ ^CD25^+ ^Foxp3^+ ^T cells (Tregs) in the spleen and MLN by flow cytometry analysis. Despite the lower Treg response in the Bry‐1‐treated *Il‐10* ^−/− ^mice compared with the WT mice, the proportion of Tregs in the Bry‐1‐treated *Il‐10* ^−/− ^mice was significantly higher than that in the untreated *Il‐10* ^−/− ^mice in both the spleen (Figure 7A,B) and the MLN (Figure 7C,D). These data indicated a promotive effect of Bry‐1 on Treg responses.

### 3.8 Effects of Bry‐1 on *Il‐10^−/−^* mice may be partly mediated via the downregulation of STAT signalling

**Figure 8:**
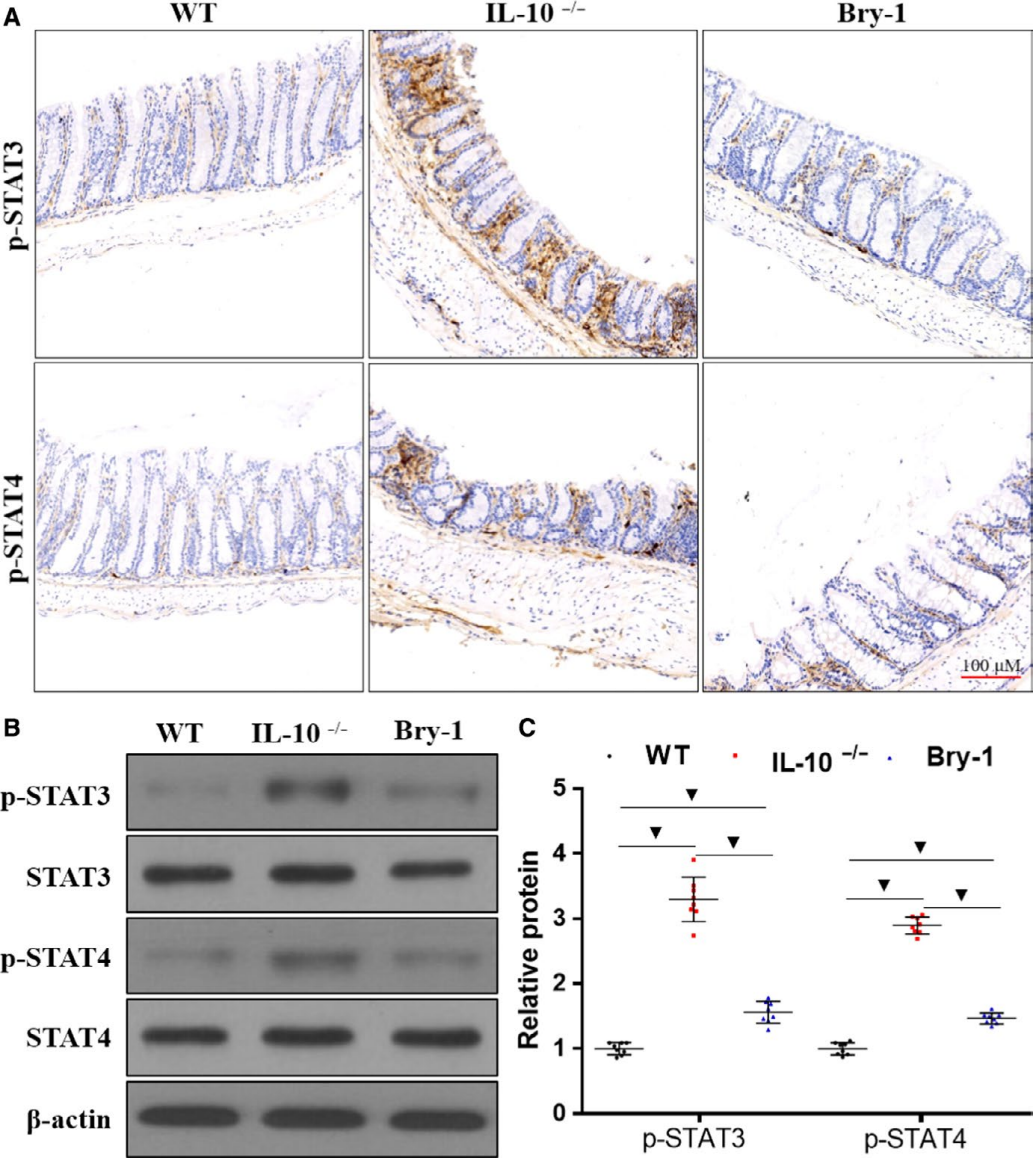
Effects of Bryostatin‐1 on *Il‐10* ^−/−^ mice may be partly mediated by downregulating STAT signalling. (A) Representative immunohistochemical staining for p‐STAT3 and p‐STAT4 is shown. Western blot analysis showed that p‐STAT3 and p‐STAT4 levels were significantly decreased in Bry‐1‐treated *Il‐10* ^−/− ^mice compared to untreated *Il‐10* ^−/− ^mice, although the levels in the Bry‐1‐treated mice were higher than those in WT mice. Bry‐1, Bryostatin‐1; WT, wild‐type; and NS, no significance. At least three independent experiments with six to eight mice in each group were performed, with one representative experiment is shown. The data are expressed as the mean ± SD. ^▼^ *P* < 0.05

Previous studies have reported that STAT signalling plays an important role in the regulation of the intestinal mucosal immune response and intestinal epithelial cell apoptosis.^17^, ^37^ We wanted to assess the effect of Bry‐1 on STAT signalling, as this information may provide a molecular explanation for the role of Bry‐1 in protecting against colitis. By immunohistochemistry (Figure 8A) and Western blotting (Figure 8B,C), we found that the p‐STAT3 and p‐STAT4 expression levels were significantly decreased in the Bry‐1‐treated *Il‐10* ^−/− ^mice compared to the untreated *Il‐10* ^−/− ^mice, although the levels in the Bry‐1‐treated mice were higher than those in the WT mice. These results may partly explain the protective role of Bry‐1 in CD‐like colitis.

## 4 DISCUSSION

Our main findings can be summarized as follows: (a) the systemic delivery of Bry‐1 ameliorated experimental colitis by attenuating intestinal barrier injury and the abnormal intestinal mucosal immune response in *Il‐10* ^−/−^ mice; and (b) the effects of Bry‐1 on spontaneous chronic colitis may be partly mediated through activating Nrf2 signalling and downregulating STAT3/4 signalling.

To the best of our knowledge, our study provides the first evidence that the administration of Bry‐1 could significantly ameliorate spontaneous CD‐like enteritis in *Il‐10* ^−/−^ mice, as demonstrated by decreases in the DAI, inflammatory score and proinflammatory mediator levels. To explore the possible mechanism underlying the antienteritis effects of Bry‐1, we analysed the changes in the intestinal barrier, as these changes are important in human CD pathogenesis. We found that Bry‐1 administration was associated with increased expression of intestinal mucosal epithelial TJ proteins (claudin‐1 and occludin). Using TEM, we found more direct evidence that Bry‐1 protected the TJ structures of intestinal mucosal epithelial cells in *Il‐10* ^−/−^ mice. Increased intestinal permeability is an important factor in causing or maintaining intestinal inflammation in CD.^6^ Our results indicated that Bry‐1 significantly decreased the intestinal permeability and bacterial translocation rates in *Il‐10* ^−/−^ mice. The therapeutic effects of Bry‐1 on CD‐like colitis and intestinal barrier dysfunction encouraged us to continue exploring. We found that intestinal mucosal epithelial cell apoptosis was decreased with Bry‐1 treatment. In addition, the increased expression of Bcl‐2 and decreased levels of Bax and cleaved caspase‐3 in the Bry‐1‐treated *Il‐10* ^−/−^ mice were confirmed. Although the changes in apoptosis‐related proteins help us to better understand the antiapoptotic effect of Bry‐1, we still wanted to discover a possible in‐depth mechanism. Activated Nrf2 signalling shows strong antiapoptotic and antioxidant effects, especially in alleviating intestinal epithelial cell apoptosis during intestinal injury in recent studies.^34^, ^35^, ^36^ In the present work, the increased expression of Nrf2 and its downstream factor HO‐1 in the Bry‐1‐treated *Il‐10* ^−/−^ mice may partly explain the protective role of Bry‐1 in *Il‐10* ^−/−^ mice.

The therapeutic effects of Bry‐1 on colitis and intestinal barrier injury in *Il‐10* ^−/−^ mice were very encouraging and suggested that Bry‐1 has potential clinical application value. These findings drove us to continue to explore the potential curative mechanisms. We evaluated the effects of Bry‐1 on the intestinal mucosal immune response and found that Bry‐1 treatment decreased the Th17 and Th1 responses and increased the Treg response in *Il‐10* ^−/−^ mice. Next, we focused on STAT signalling, as this pathway has a key role in regulating T cell‐mediated immune responses in human CD.^38^ We found that p‐STAT3 and p‐STAT4 expression decreased with Bry‐1 treatment in *Il‐10* ^−/−^ mice. Activated STAT4 is important for the differentiation of Th1 cells, and activated STAT3 is important for the differentiation, functions and amplification of Th17 cells.^39^, ^40^ In addition, the inhibition of STAT3 contributes to the activation of Tregs.^41^ These findings may partly explain the effects of Bry‐1 on the intestinal mucosal immune response in CD‐like colitis.

The findings of our study have potential clinical implications. Given the rising incidence of CD and the importance of drug therapy, research teams, including our team, are looking for possible new drugs. However, drugs that have the potential to be applied in the clinic soon may be more valuable than those that will still require extensive research. In fact, to the best of our knowledge, Bry‐1 has been studied for more than 30 years.^42^ Previous studies have shown that Bry‐1 has immunomodulatory, anti‐oxidant and anti‐inflammatory effects, suggesting its potential clinical application value.^19^ Although the biological functions of Bry‐1 have not been fully revealed, in the past 10 years, more than 20 clinical trials have been conducted with Bry‐1 as a monotherapy or in combination with clinically used cytotoxic drugs.^43^ A recent clinical trial confirmed the efficacy and safety of Bry‐1 in the treatment of Alzheimer's disease.^21^ Even more exciting findings in recent research have shown that Bry‐1 has therapeutic potential in progressive forms of multiple sclerosis, which have immune response types similar to those of CD.^20^ We hope that Bry‐1 may benefit CD patients in the future.

Our study has some limitations. For example, our results showed that Bry‐1 protects against CD‐like colitis by improving the intestinal barrier and the abnormal intestinal mucosal immune response; however, Bry‐1 could also improve colitis through other means. The changes in Nrf2 and STAT3/4 signalling may partly explain the mechanism underlying the treatment effects of Bry‐1, but we may have ignored other signalling pathways. It seems likely that Bry‐1 has multiple biological functions, as previously reported.^19^

In conclusion, this study provides initial evidence that the systemic delivery of Bry‐1 ameliorates spontaneous colitis in *Il‐10* ^−/−^ mice, and this effect is associated with the attenuation of intestinal barrier injury and the abnormal intestinal mucosal immune response. The protective effect of Bry‐1 on CD‐like colitis, particularly given the established clinical safety of Bry‐1, suggests Bry‐1 will have therapeutic potential in human CD.

## CONFLICT OF INTEREST

The authors declared no financial conflict of interest.

## AUTHOR CONTRIBUTIONS

J. Li, L. Zuo and S. Ge contributed to the study concept and design, data acquisition, experiments, data analysis and manuscript drafting. J. Hu designed the experiments. M. Shen, C. Zhou and Y. Wang contributed to the animal experiments and testing. Y. Ge and R. Wu contributed technical support and scientific advice and helped with manuscript revision. All authors read and approved the final manuscript.
